# Beyond Hypothesis Testing: Investigating the Diversity of Scientific Methods in Science Teachers’ Understanding

**DOI:** 10.1007/s11191-020-00185-9

**Published:** 2021-02-06

**Authors:** Olga Ioannidou, Sibel Erduran

**Affiliations:** Department of Education University of Oxford, 15 Norham Gardens, Oxford, OX2 6PY UK

## Abstract

Recent reforms in science education have promoted students’ understanding of how science works, including the methodological approaches used by scientists. Given that teachers are expected to teach and promote methodological pluralism, it is worth examining how teachers understand and view scientific methods, particularly when scientific methods are presented as a diverse array and not as a linear model based exclusively on hypothesis testing*.* The empirical study presented in the paper examines science teachers’ understanding of scientific methods, particularly the diversity of scientific methods. Brandon’s Matrix, a philosopher’s account of scientific methods, has been adapted for educational purposes, and two tasks were developed in order to investigate teachers’ understanding of scientific methods. Fifty-six science teachers (25% male, 75% female) from different regions in the UK responded to an online survey*.* The results showed that the majority of the teachers showed satisfactory understanding of basic components of Brandon’s Matrix. However, more than half of the sample held naïve understanding of scientific methods. By providing insight into teachers’ misconceptions about scientific methods, the study provides suggestions for the design of teacher training programmes and highlights the need for explicit instruction about scientific methods. In addition, we suggest the use of heuristics such as Brandon’s Matrix for the development of pedagogical tools as well as research instruments.

## Introduction

Research on the teaching and learning of the scientific method has a long history (Blachowicz 2009; McPherson 2001). The conceptualisation of the “scientific method” in school science is often based on a standard method involving a specific procedure. Scientific method is meant to involve testing of a hypothesis through a careful consideration of independent and dependent variables. The scientific method has been widely used as a term to encapsulate the methods that scientists follow “for testing ideas and theories” (Merriam-Webster Dictionary n.d.). These definitions represent scientific methods as a step-wise process through which scientists produce robust evidence by applying specific practices and methods, such as experimentation and observation.

Having reviewed 70 introductory science textbooks, Blachowicz (2009) illustrated that textbooks tend to present the scientific method as a stepwise process in a simple empiricist view of science. Woodcock (2014) discussed such “myths” of the scientific method and highlighted a wide variation in the content of scientific method representations that range from 2–3 steps to 11 steps. These steps may include the following scientific processes: observing, making a hypothesis, experimenting, analysing data, confirming or rejecting the hypothesis and making conclusions. Such generic steps have also been challenged due to variations in domain-general versus domain-specific approaches to scientific methods (e.g. Frodeman 1995). For example, Dodick et al. (2009) highlighted the importance of distinguishing scientific methodology in the historical and experimental sciences. Science disciplines like geology, evolutionary biology or cosmology, as described by many (Cleland 2013; Dodick et al. 2009; Frodeman 1995; Rudolph 2005; Turner 2013), have highlighted that the perception of the hypothetico-deductive reasoning being the best or only way in which scientific knowledge develops is misguided.

Teachers as mediators of the science curriculum (Joyce et al. 2003) can potentially play an active role in incorporating the idea of methodological pluralism in their teaching practices. However, in order to teach “how science works”, teachers often appeal to the paradigm of “the scientific method” (Woodcock 2014) and use the popular depiction of the scientific method as a linear stepwise process given the prominence of this model in science textbooks. Numerous studies have highlighted the shortcomings of positioning the scientific method in such a simplistic fashion as a linear process (e.g. Erduran and Dagher 2014; Kind and Osborne 2017). One point of criticism refers to the false portrayal of science as a linear and straightforward process, and how this portrayal cultivates student misconceptions about “what science is” and “how it works” (Mccomas 1998). Furthermore, the emphasis of methodological approach being biased towards experimentation and hypothesis testing, implies that these methods are the epitome of scientific rigour (Kind and Osborne 2017). By limiting the range of scientific methods used in science classrooms and by excluding scientific methods and practices that do not fall into these categories, students’ understanding of scientific aims and methods can be limited (Dagher and Boujaoude 2005; Driver et al. 1996; Schwartz 2007).

In this paper, we problematise the notion of the singular and simplistic characterisation of the “scientific method” in the context of science education and present an investigation about how teachers differentiate between scientific methods and whether they view “the scientific method” as the universal method that scientists follow. Capitalising on a framework based on the work of philosopher Brandon (1994), we illustrate how scientific methods can be diversified in the context of science teaching and learning. Hence, the paper uses a theoretical framework from a philosopher’s account in order to attain conceptual clarity about the characterisation of scientific methods. In the empirical component of the paper, we use Brandon’s framework in order to investigate teachers’ understanding and views of scientific methods. The study investigated science teachers’ understanding and nature of science (NOS) views regarding scientific methods. Hence, Brandon’s framework is also serving as a tool in designing an analytical approach in order to investigate teachers’ understanding and views. The findings of the study can help inform future research and development about how to include scientific methods in teacher education. In addition, the study provides insight on the use of theoretical frameworks, such as Brandon’s Matrix for the design and analysis of tasks measuring understanding of NOS.

## Scientific Methods and Science Education

Science curricula have often utilised “the scientific method” as a tool to schematically illustrate the specific steps that scientists follow within their investigations. Curricula, textbooks and, subsequently, teachers have promoted the idea of “the scientific method” as a unique step-wise process through which scientists test their hypotheses and draw conclusions. A popular representation of the scientific method (e.g. GeneseeChemistry n.d.) includes the following steps:

1. Scientific investigations start with a specific question.
2. Scientists conduct background research by reviewing the relevant literature and other sources of evidence related to the question.
3. Based on this background research, a hypothesis is constructed, which is tested by an experiment.
4. The collected data is analysed in order to draw conclusions.
5. Scientists communicate their findings by revising their initial hypothesis.

This model has proved itself appealing for teaching purposes, as it represents a simple and reproducible process through which students can plan, carry out and communicate scientific investigations. Woodcock (2014) summarises five potential functions of the scientific method: informative, prescriptive, participative, demarcative and elevative. It is informative because it illustrates “how science works” (p.7) and prescriptive because it conveys to students the idea that scientific evidence is produced by following a straightforward method. It is participative because it enables students to play the role of a scientist. It is demarcative because it prescribes a set of criteria for scientific processes (e.g. the investigation of a question or the testing of a specific hypothesis). Finally, it is elevative as it represents the decisions that were made during the scientific investigations as rational, objective and logical.

In support of the criticism against “the scientific method”, previous research has demonstrated that scientists use a plethora of methods and tools to produce scientific evidence (e.g. Bauer 1994). The study of methods applied in science unravels a variety of methods applied across different domains, as well as within the same domain. In sciences such as astronomy, scientists often apply observational methods to gather information about celestial bodies with use of telescopes. Although there is no manipulation of variables involved in these investigations, the methods that are used are legitimate and rigorous for gathering information and provide evidence for answering given questions. The choice of methods often depends on the nature of the question that scientists pose, as well as the availability of tools and methods in a given time. For instance, when astronomers want to answer questions about the characteristics of distant planets, they cannot manipulate the objects or change any of their characteristics. Yet, even if they could, this would not be necessary to answer their question. Thus, Erduran and Dagher (2014) argue: “The discrimination of adequate tools and proper methods falls under the purview of domain-specific experts and it is not a matter of public opinion”(p.96).

 In order to represent scientific methods in school science in a manner that is authentic to science, teachers and students need to be exposed to the different methods with which science is conducted. In so doing, school science would enable teachers and students alike to appreciate the different methods through which data are obtained, for instance by considering the different scientific cultures and traditions in which the methods have evolved. In this regard, science education can promote the idea that it is not one method or a specific line of evidence that can support theoretical claims in science, but rather, it is the convergence of evidence from various sources that lead to scientific explanations (Erduran and Dagher 2014).

### Articulating the Diversity of Scientific Methods

The conceptualisation of “scientific methods” has often centred in debates about the nature of science (e.g. Allchin 2011; Irzik and Nola 2014; Thagard 2012). There have been some attempts to represent a broader range of scientific methods, by frameworks alternative to “the scientific method” (e.g. Lawson 2003; Rudolph 2005; Turner 2013). For instance, Wivagg and Allchin (2002) proposed the “scientists’ toolbox” to highlight the different directions that scientific investigations can take. In conceptualising the nature of science, Irzik and Nola (2011, 2014) as well as Erduran and Dagher (2014) have emphasised the importance of facilitating understanding of the complexity of scientific methods beyond the singular and linear “scientific method”. Numerous studies have articulated the domain-general versus domain-specific approaches to scientific methods (e.g. Frodeman 1995). For example, Dodick et al. (2009) highlighted the importance of distinguishing scientific methodology in the historical and experimental sciences.

Brandon’s Matrix is an example framework that has been investigated recently both theoretically (Erduran and Dagher 2014) and empirically (e.g. Cullinane et al. 2019; El Masri et al. 2021). Erduran and Dagher (2014) proposed the use of Brandon’s Matrix as a useful tool to illustrate the variety of scientific methods. Brandon (1994) proposed an integrative framework representing scientific investigations based on whether (a) they include the manipulation of a variable and (b) they involve hypothesis testing. Based on these two parameters, scientific investigations can be grouped in four categories presented in a two-by-two table represented here with examples suggested by Erduran and Dagher (2014) (Table 1).

**Table 1:** Representation of scientific methods (reproduced from Erduran and Dagher 2014, p. 101)

	Experiment/Observation	
	Manipulate	Not manipulate
Test hypothesis	Manipulative hypothesis test e.g. *Investigations in Genetics-molecular evolution*	Non-manipulative hypothesis test e.g. *Observation of Darwin’s finches*
Measure parameter	Manipulative description or measure e.g. *Artificial selection and breeding*	Non-manipulative description or measure e.g. *Studies in palaeontology and developmental biology*

Brandon’s Matrix depicts the breath of scientific methods, without imposing a hierarchy between the methods. In this way, experimentation (or the manipulation of a variable) and hypothesis testing are presented as possibilities rather than necessities for a scientific investigation. An investigation can be experimental without involving hypothesis testing, while there are investigations that do not include the manipulation of a variable or hypothesis testing. Brandon gives the example of artificial selection and breeding as methods that include the manipulation of variable(s) but do not involve hypothesis testing (Table 1). Similarly, Brandon’s Matrix presents the example of palaeontology and developmental biology as scientific areas in which investigations do not include the manipulation of a variable, nor the testing of a hypothesis. This representation can be used to illustrate the diversity in methods in specific science domains. For instance, Erduran and Dagher (2014) presented examples from chemistry by drawing examples from Scerri’s work (2007), who described Mendeleev’s predictions about the existence of gallium (or eka-aluminium) with the use of non-manipulative description and quantitative reasoning about weights.

**Fig. 1:**
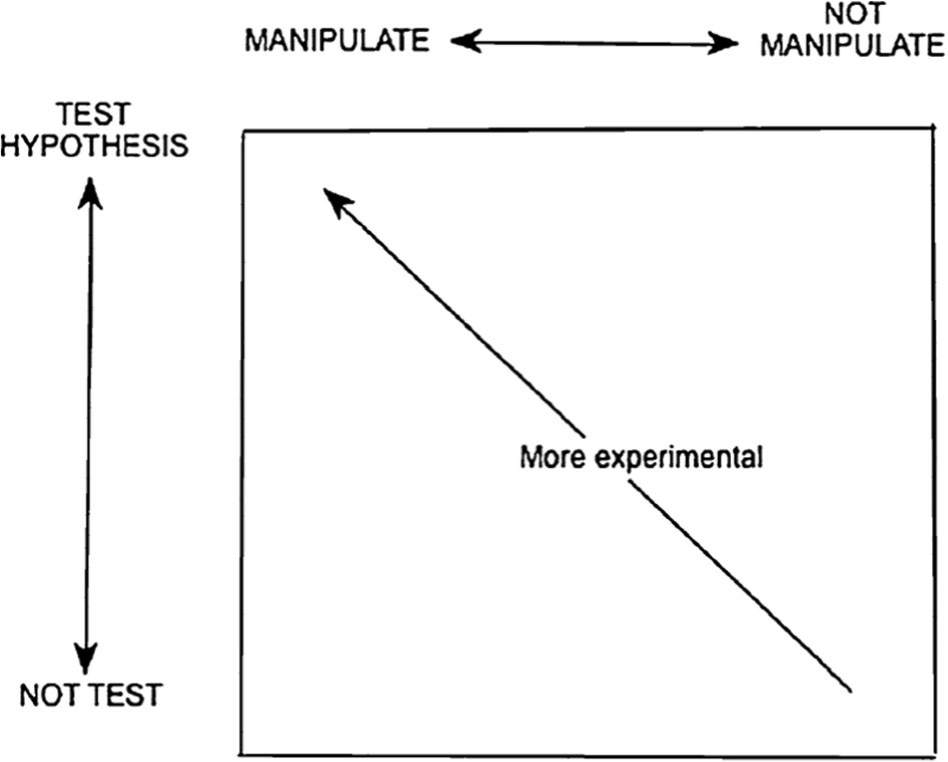
Brandon’s representation of “space of experimentality” between two continua (from Brandon 1994, p.66)

While Brandon’s Matrix is represented in its simplistic form as a 2-by-2 table, it is indeed a more complex model that accounts for the observation that methods in science are rarely clear cut (e.g. Allchin 2005; Lawson 2003, 2006). While methods can be conceptualised as dichotomies, Brandon (1994) states that it is useful to view them as components of two continua that range from testing to not testing and from manipulation to non-manipulation. A given branch of science can utilise a continuum of methods. He represents this relationship in the way depicted in Fig. 1 whereby investigations can be viewed as more (upper left corner) or less (lower right corner) experimental.

As a theoretical framework, Brandon’s Matrix is simple enough to illustrate that scientific method is not singular nor linear. As such it offers flexibility in its utility as an analytical tool. For example, Cullinane et al. (2019) used Brandon’s Matrix to show what methods underlie the practical chemistry items in high-stakes examination papers. The authors illustrated how manipulative parameter measurement dominated the examination papers and how manipulative hypothesis testing type questions were present in a limited capacity. This was contrary to initial belief that manipulative hypothesis testing would be dominant, as this is often presented as “the scientific method” in many science classrooms around the world. This inconsistency between the well-established “scientific method” and the presentation of science methods in high-stakes examination is a recipe for confusion, as well as leading to cookbook style procedures in the science classroom. The study therefore showed there is a disconnect with what is being presented as methods in science, the methods they are performing to draw conclusions from investigations, and the methods of practical science appearing on examination materials.

As a pedagogical tool, Brandon’s Matrix can be used to promote the idea that science does not follow one specific method and that scientific evidence is produced by the synergies between various methods and scientific fields. Erduran and Kaya (2018, 2019) used Brandon’s Matrix to improve pre-service teachers’ understanding of scientific methods through visual representations. The matrix can be used as a metacognitive tool, as it gives the opportunity to teachers and students to reflect on the scientific investigations that students undertake in science classrooms. During this process, they would discover that the categorisation of the scientific investigations into Brandon’s Matrix is not rigid, as the matrix can be also viewed as a continuum between “more experimental” and “less experimental” investigations (Erduran and Dagher 2014). This way teachers and students would engage in more “minds-on” activities, as they would have to reflect on the methods that they use and the decision-making processes that take place before the application of the methods.

However, the application of heuristics, such as Brandon’s Matrix, as pedagogical tools depends on the degree to which teachers understand and view these tools. Prior research has highlighted the importance of teachers’ nature of science (NOS) views and their impact on their teaching practices, based on the argument that teachers are not able to teach NOS unless they—themselves—hold informed NOS views (Capps et al. 2012).

### Teachers’ Views of Scientific Methods

The understanding of scientific methods and the appreciation of methodological pluralism in science has been studied as part of nature of science (NOS) views (e.g. Schwartz and Lederman 2008; Schwartz et al. 2008) and nature of scientific inquiry (Schwartz et al. 2012). However, researchers have noted that there is no one scientific method; there has been little concrete articulation of what the plurality involves. Furthermore, although teaching about the nature of science and scientific methods has been emphasised by curricula, recent studies have shown that teachers report difficulties in teaching nature of science (Kampourakis 2016). These difficulties often derive from teachers’ naïve views about NOS and misconceptions about what science is and how it operates (Akerson et al. 2009; Dogan and Abd-El-Khalick 2008; Guerra-Ramos et al. 2010). There has been substantial literature reporting teachers’ lack of understanding of scientific inquiry and NOS (e.g. Abd-El-Khalick and BouJaoude 1997; Carey and Stauss 1970; Pomeroy 1993).

In a study investigating teacher practices regarding NOS, Capps and Crawford (2013) showed that the 26 participants held naive views about inquiry-based instruction, as well as NOS, which were reflected in their teaching practices. In a recent study, Kartal et al. (2018) investigated the impact of a continuous professional development (CPD) program that promoted collaboration and reflection on teachers’ NOS views. The results showed that the majority of the teachers held naïve views about scientific methods before their participation in the CPD program. More explicitly, the study reported that 72% of the participants (*N* = 18) believed that “there is a single universal method which scientists follow step-by-step to reach conclusions” (p. 5). Teachers’ views regarding scientific methods were significantly improved after the completion of the CPD program, highlighting that teachers’ views about scientific methods were the most amendable among other NOS aspects (e.g. nature of scientific theories and laws) (Kartal et al. 2018).

Similarly, Zion et al. (2018) conducted a fine-grained analysis on teachers’ conceptions about NOS and scientific inquiry identifying three themes with regard to teachers’ understanding of scientific methods: “1) distinguishing an observation from an experiment, 2) mapping observation as a scientific method and 3) acknowledging that there are various scientific methods” (Zion et al. 2018, p. 15). In addition, the study concluded that teachers’ views on scientific methods can be improved with the use of open inquiry and personal inquiry approaches.

Despite a wealth of studies on teachers’ understanding of related themes such as scientific inquiry and NOS, there is a paucity of research on how teachers make sense of different accounts of scientific methods. For example, there is limited understanding of how science teachers consider the role of experimental design in relation to hypothesis testing and whether or not an experiment is a necessary component of the scientific method. In addition, only a few of these studies have explicitly measured teachers’ understanding of scientific methods (e.g. Kartal et al. 2018). Previous research has shown that teachers’ views on scientific methods are among the NOS aspects that can be improved through CPD programmes (Kartal et al. 2018; Zion et al. 2018). Nevertheless, in order to improve teachers’ views, teacher training programmes should be informed by teachers’ prior understanding of scientific methods. Hence, the description in the empirical part of the paper will focus on an investigation into secondary science teachers’ understanding of the diversity of scientific methods and the components of these methods as specified by a characterisation based on Brandon’s (1994) framework.

## Methodology

### Research Questions

Considering the arguments advocated by major curriculum standards documents such as the *Next Generation Science Standards* (e.g. NGSS Lead States 2013) that science teaching should reflect authentic NOS, it is vital to develop science teachers’ understanding of how different scientific methods contribute to doing of science. The policy recommendations imply that teachers’ understanding of science needs to go beyond the mythical linear depiction of the scientific method to be more inclusive of a diversity of methods. Thus, the research questions addressed in this study aimed to address contemporary calls for advancing teachers’ understanding of science. These calls target some fundamental questions about how science teachers understand scientific methods and their diversity:

1. What is science teachers’ understanding of scientific methods?
2. How do science teachers view the diversity of scientific methods?

Although both of our questions address teachers’ overall perception of scientific methods, each research question refers to a distinct construct. The difference between “understanding” and “views” of scientific methods as used in this study is highlighted to clarify their particular nuance in relation to the research questions.

In our first research question, we use the term “understanding” of scientific methods to describe teachers’ ability to identify the basic components of scientific investigations as commonly presented in classrooms. As the next section on the research context illustrates, the science curriculum in our educational context requires teachers to include in their lessons themes related to scientific methods. Therefore, we aimed to examine whether teachers can recognise (a) hypothesis testing and (b) the manipulation of variables when they are present in examples of practical investigations. Thus, as presented in the section describing the designed instruments, the task that was designed to address teachers’ understanding of scientific methods would resemble multiple choice knowledge tests, containing pre-defined correct answers.

In our second research question, we use the term “views” to describe teachers’ NOS views relevant to scientific methods. More explicitly, “views” refer to teachers’ perceptions regarding the linearity and universality of scientific method(s), as well as the relative significance of specific scientific methods (hypothesis testing and variable manipulation). This construct includes teachers’ general views regarding science and scientific methods that are often subjective. Thus, contrary to understanding of scientific methods, teachers’ views cannot be assessed on the basis of their correctness, but rather on the basis of their sophistication. The study is framed by Brandon’s (1994) Matrix in its theoretical characterisation of scientific methods which has served to develop the research instruments to address the research questions.

### Research Context

The study was conducted in England where the use of the terminology “practical science” has been widely used in the curriculum, exam board specifications and research traditions in science education for several decades (Erduran et al. 2020). For example, the Royal Society has used the terms “practical science” as “…a shorthand for the full programme of experimental and investigative activities (including fieldwork) conducted as part of science education in schools and colleges” (House of Lords 2006, p. 63).

The Office of Qualifications and Examinations Regulation advocates that at GCSE level (ages 14–16) pupils should “develop their ability to evaluate claims based on science through critical analysis of the methodology, evidence and conclusions, both qualitatively and quantitatively” (Ofqual 2015, p.5). Ofqual is a non-ministerial government organisation that regulates qualifications, exams and tests in England. Each GCSE qualification is in a particular subject such as biology, physics and chemistry and stands alone (although a set can also be pursued). Studies for GCSE examinations generally take place over 2 or 3 years depending on the subject. Science subjects no longer include a “hands-on” assessment of practical science skills as they did pre-reform; instead, the final exam papers are intended to have items specifically written to indirectly assess students’ knowledge and understanding of practical science (Ofqual 2015).

The science curriculum at GCSE level contains a component referred to as “Working Scientifically”. In advancing “Working Scientifically” as a curricular goal for GSCE science, Department for Education (2014) has put forward four key areas of skills to be developed: (a) development of scientific thinking, (b) experimental skills and strategies, (c) analysis and evaluation and (d) scientific vocabulary, quantities, units, symbols and nomenclature (p. 7–8). The coverage of scientific methods is thus advocated in the context of related concepts by both Ofqual and the Department of Education.

### Instruments

In order to measure teachers’ understanding of scientific methods, a survey of nine items was developed based on the theoretical ideas about scientific methods underlined by Brandon’s Matrix. The choice of Brandon’s Matrix was decided for two reasons. First, Brandon’s Matrix provides a holistic overview of scientific methods and as such, it can serve as a tool to examine teachers’ ability to identify different types of scientific methods (e.g. hypothesis/no hypothesis testing). Second, because of its non-hierarchical nature, Brandon’s Matrix can be used to examine teachers’ whether or not teachers are inclined to consider scientific methods to be more or less significant in science.

The use of heuristics as tools for the development of research instruments has been highlighted in previous research. For instance, Chamizo and García-Franco (2013) used heuristic diagrams to design task in order to assess teachers’ research skills. They argued that heuristics can be useful tools for the design of tasks because they help identifying all elements included in the investigation (Trowbridge and Wandersee 1998). While Brandon’s Matrix does not include related aspects of scientific methods such as analogical reasoning (e.g. Duit et al. 2001) and visualisation (e.g. Gilbert 2010), it does provide a tangible framework to explore how teachers view the diversity of scientific methods. Because Brandon’s Matrix is a typology illustrating a meta-perspective on types of scientific methods, future studies can potentially focus on more nuanced features such as how some hypotheses might involve analogical reasoning in problem solving (e.g. Bancong and Song 2020).

Deriving from the same argument, the tasks of the present study were designed with regard to two aspects: (a) the understanding of the components of Brandon’s Matrix (Task 1) and (b) NOS views underlined in Brandon’s Matrix (Task 2). We included these two aspects based on the assumption that understanding of the basic elements of the Matrix would be the basis for the development for a deeper understanding of scientific methods.

#### Task 1: Understanding of the Components of Brandon’s Matrix

The basic components of Brandon’s Matrix are hypothesis testing and manipulation of a variable. Therefore, one of the aims of Task 1 was to evaluate teachers’ understanding of these two elements. In doing so, teachers categorised two scenarios of classroom investigations into one of the four categories of Brandon’s Matrix (Table 2).

**Table 2:** Question items and responses for Task 1

Item	Scenario	Multiple choice response
1.1	*Pupils want to answer the following question: “What are the relative ages of some features of the Moon?”. To answer their question, they observe four images of the Moon to compare craters and their density*	a) manipulation of one or more variables to test a hypothesis b) observation of one or more variables to test a hypothesis c) manipulation of one or more variables to describe a characteristic d) observation of one or more variables to describe a characteristic
1.2	*Pupils want to determine the best windmill design by developing and testing windmills made from a variety materials. For this investigation they use multimeters, generators, wires, PVC pipes, fabric and other building materials. Their idea is that the lighter the material the faster the blade rotation. For every new material that they use, they measure the difference in wind speed and blade rotation.*	

Although various examples of investigations can be found in science textbooks, we decided to use examples that represented the two “extreme” categories of Brandon’s Matrix (the manipulative hypothesis testing and the non-manipulative parameter measurement), as it was assumed that these categories would be easier for teachers to identify. The selected scenarios were examples of student investigations and were presented in previous research (Binns and Bell 2015), as representative examples of experimental and non-experimental investigations. The two scenarios, as well as the multiple-choice answers, are presented in Table 2. While item 1.1 has a more science orientation, item 1.2 is applied in the context of technology. However, while the ultimate purpose of investigations might be different in science versus technology (for instance, understanding/explanation in science versus maximising a result in technology), the pattern of reasoning is similar in terms of this particular aspect of hypothesis testing and parameter manipulation. Similar to the science versus technology distinction, potential other tasks would likely have nuances that might differentiate them relative to other factors as well. For instance, some tasks might have more relevance to everyday life than others. Through task design, we are not making an argument that the task components are all of the same kind but rather that the tasks utilise similar reasoning strategies that appeal to Brandon’s categories along with any other aspects that might set them as being different types of tasks as well.

#### Task 2: Nature of Science Views Underlined in Brandon’s Matrix

The second task consisted of seven Likert-scale questions that aimed to examine teachers’ views on scientific methods. Three of these items targeted teachers’ general views on scientific methods, while four aimed to measure teachers’ views regarding the scientific methods presented within Brandon’s Matrix. Table 3 presents the items and the targeted constructs.

**Table 3:** Questions in Task 2 and their targeting constructs

Item	Construct
2.1 There is a universal scientific method which scientists follow.	VOSM general
2.2 In science investigations follow step-by-step procedures.	VOSM general
2.3 An experiment is not always the best way to test a hypothesis.	VOSM (BM-specific)
2.4 In order to be scientific, an investigation should include hypothesis testing	VOSM (BM-specific)
2.5 In order to be scientific, an investigation should include manipulation of a variable.	VOSM (BM-specific)
2.6 Observations that do not include hypothesis testing or a manipulation of a variable are not scientific investigations.	VOSM (BM-specific)
2. 7 Science does not always follow a universal method.	VOSM general

The items that were used to measure teachers’ general views were adapted items from previous research. More explicitly, Item 2.1 (“There is a universal scientific method which scientists follow.”) was adapted by a scoring rubric previously used by Kartal et al. (2018). Similarly, Item 2.2 (“In science investigations follow step-by-step procedures.”) was previously used by Lederman et al. (2014) (VOSI, Item 3c). These items were selected because, in line with Brandon’s Matrix, they target teachers’ views of “the scientific method”.

The items targeting teachers’ views regarding the scientific methods presented in the matrix were developed for the needs of the present study. More explicitly, the items aimed to capture teachers’ views on the two basic components of Brandon’s Matrix (hypothesis testing and manipulation of a variable) (Items 2.4 and 2.5), as well as their views on the two “extreme cases” of scientific investigations (manipulative hypothesis testing and non-manipulative parameter measurement) (Items 2.3 and 2.6). This would allow comparisons between their understanding of the components of the matrix (Task 1 and Task 2).

Both of the tasks were reviewed by a team of experts in an iterative process. One of the authors is an expert on NOS and two researchers are also science teachers who reviewed the items for clarity and difficulty. Face validity of the items was established through a review process involving a subject matter expert.

### Participants

The sample consisted of 56 science teachers (25% male, 75% female) from different regions in the UK. The participants were teachers recruited as a part of a CPD program and they completed the designed tasks before the beginning of the training. Participants took part in the program, as well as the study, voluntarily. Therefore, the study followed the voluntary response sample method. The training was designed and delivered in the context of Project Calibrate (projectcalibrate.web.ox.ac.uk), which aimed to introduce Brandon’s Matrix as a pedagogical tool for teaching and assessment of practical science at the GCSE level (Erduran 2020).

**Fig. 2:**
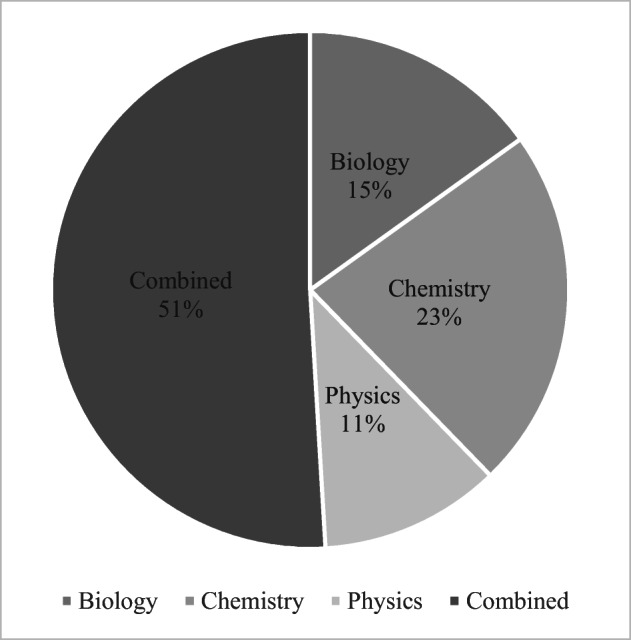
Percentages of participants by teaching subjects (*N* = 56)

Fifty-five out of 56 teachers were active in-service teachers, while their average teaching experience was 8.5 years *(SD* = 6.89). The percentages of their teaching subjects are displayed in Fig. 2. The category “combined” refers to teachers that taught more than one science subject.

### Data Analysis

#### Task 1: Understanding of the Components of Brandon’s Matrix

As described in the previous section, the aim of Task 1 was to evaluate teachers’ understanding regarding the concepts of hypothesis testing and manipulation of variables. To enable the identification of misconceptions, as well as variance between the answers, the multiple-choice answers were partially graded. Thus, participants received points for every correct element that their answers contained. Participants received two points for every correct identification of variable manipulation and one point for identifying hypothesis testing. This was decided because hypothesis testing could be falsely assumed even though it was not mentioned in the given scenarios. Therefore, the grading rubric prioritised variable manipulation, while it favoured the correct answer with an additional point. For every item the maximum score was four (4) and the minimum zero (0). Table 4 displays the points assigned for each of the elements of the multiple-choice answers.

**Table 4:** Scoring rubric for Task 1

	Answer		Elements included	Points	Total
Item 1.1	1a		Manipulation	0	0
			Hypothesis	0	
	1c		Manipulation	0	1
			Parameter	1	
	1b		Observation	2	2
			Hypothesis	0	
	1d	(correct answer)	Observation	2	4
			Parameter	2	
Item 1.2	2d		Observation	0	0
			Parameter	0	
	2b		Observation	0	1
			Hypothesis	1	
	2c		Manipulation	2	2
			Parameter	0	
	2a	(correct answer)	Manipulation	2	4
			Hypothesis	2	

#### Task 2: Nature of Science Views Underlined in Brandon’s Matrix

To evaluate teachers’ responses with regard to their nature of science views, the five-point Likert scale answers were scored based on whether the answer reflected informed or naïve nature of science views. The scores ranged between 1 (more naïve views) and 5 (more informed views). The scoring rubric applied for Task 2 is presented in Table 5.

**Table 5:** Scoring rubric for Task 2

Item	Answers				
	(More naïve views)		(More informed views)		

2.1 There is a universal scientific method which scientists follow.	SA	A	N	D	SD
2.2 In science investigations follow step-by-step procedures.	SA	A	N	D	SD
2.3 An experiment is not always the best way to test a hypothesis.*	SD	D	N	A	SA
2.4 In order to be scientific, an investigation should include hypothesis testing	SA	A	N	D	SD
2.5 In order to be scientific, an investigation should include manipulation of a variable.	SA	A	N	D	SD
2.6 Observations that do not include hypothesis testing or a manipulation of a variable are not scientific investigations.	SA	A	N	D	SD
2. 7 Science does not always follow a universal method.*	SD	D	N	A	SA
Scores	1	2	3	4	5

After the evaluation of participants’ responses, the frequencies of teachers’ answers were calculated. In addition, in order to examine potential patterns in the given answers, inter-item correlations were calculated. The results are presented in the next section.

## Results and Findings

This section summarises the key results and findings from the two tasks related to teachers’ understanding of the components of Brandon’s Matrix and their NOS regarding scientific methods.

### Task 1: Understanding of the Components of Brandon’s Matrix

**Fig. 3:**
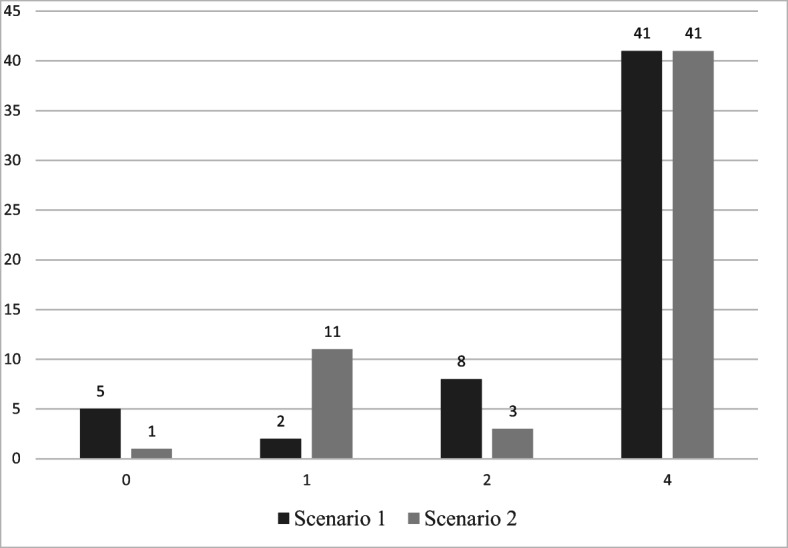
Frequencies of teachers’ scores on understanding of the components of Brandon’s Matrix (*N* = 56)

To answer our first research question, teachers were asked to categorise two scenarios of student investigations based on whether they use hypothesis testing and/or manipulation of the variables. The results showed that 73% (*N* = 56) of the teachers selected the correct answer in both of the scenarios. Figure 3 displays the scores for Task 1 in more detail.

### Task 2: Nature of Science Understanding and Brandon’s Matrix

With regard to the second research question, teachers were asked to answer seven items of in a 5-point Likert scale. The reliability of the instrument was calculated through inter-item correlations. This decision was made as inter-item correlations are widely performed for scales with a small number of items in order to examine the degree to which scores on one item are related to the rest of the items included in the scale. Thus, inter-item correlations are used as a measurement of item relatedness, as well as item redundancy (Cohen and Swerdlik 2005).

Table 6 and Table 7 present the inter-item correlations per sub-scale (VOSI general and BM specific items). As presented in the tables, the inter-item correlations ranged between .20 and .50 for all items, with the exception of Item 2.3. This means that the sub-scales were reasonably homogenous and not isomorphic with each other (Piedmont 2014). One possible explanation for the low values of Item 2.3 is that it was worded negatively; therefore, participants demonstrated a different response pattern when answering the item. Another explanation could be that the item is not representative of the same sub-scale; thus, the sub-scale is not unidimensional.

**Table 6:** Inter-item correlations for VOSM-general items

	2.1 There is a universal scientific method which scientists follow.	2.2 In science all investigations follow step-by-step procedures.	2.7. Science does not always follow a universal method. *(reverse)*
2.1 There is a universal scientific method which scientists follow.	1.00	.36	.25
2.2 In science all investigations follow step-by-step procedures.	.36	1.00	.37
2.7. Science does not always follow a universal method. *(reverse)*	.25	.37	1.00

**Table 7:** Inter-item correlations between items referring to Brandon’s Matrix (VOSM-BM specific)

	2.4 In order to be scientific, an investigation should include hypothesis testing.	2.5 In order to be scientific, an investigation should include manipulation of a variable.	2.6 Observations that do not include hypothesis testing or a manipulation of a variable are not.	2.3 An experiment is not always the best way to test a hypothesis.
2.4 In order to be scientific, an investigation should include hypothesis testing.	1.00	.46	.20	.13
2.5 In order to be scientific, an investigation should include manipulation of a variable.	.46	1.00	.49	.23
2.6 Observations that do not include hypothesis testing or a manipulation of a variable are not.	.20	.49	1.00	.04
2.3 An experiment is not always the best way to test a hypothesis.	.13	.23	.04	1.00

**Fig. 4:**
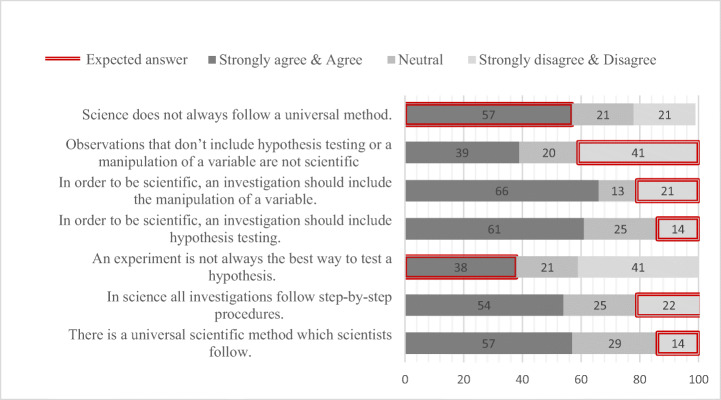
Percentages of teachers’ scores regarding NOS (*N* = 56)

The descriptive statistics indicated that 57% of the teachers agreed or strongly agreed that “There is a universal scientific method that scientists follow”, while 54% agreed or strongly agreed that “In science all investigations follow step-by- step procedures”. In addition, more than half of the sample (61%) agreed or strongly agreed that “In order to be scientific, an investigation should include hypothesis testing”. Similarly, 66% of the sample agreed or strongly agreed that in order to be scientific, an investigation should include the manipulation of a variable. The percentages of teachers’ answers are presented in Fig. 4. For illustrative reasons, Fig. 4 also presents the answers that teachers were expected to select. As described in the introduction, given that teachers are expected to teach a variety of scientific methods, their answers were anticipated to lean towards more informed NOS views.

In addition, to examine the relationship between teachers’ understanding and their views on scientific methods, Pearson’s correlation was calculated between the mean scores for Task 1 and each of the items in Task 2. The results showed that the mean scores for Task 1 were significantly positively correlated with Item 2.4 (“In order to be scientific, an investigation should include hypothesis testing”) r(55) = .30, *p* < .05 and Item 2.5 (“In order to be scientific, an investigation should include manipulation of a variable”) r(55) = .35, *p* = .008.

## Discussion and Implications

The paper was guided by the observation that school science often presents the scientific method as a simplistic and linear process, not taking into account the diversity of methods used in science. In order to investigate how science teachers view scientific methods, we appealed to Brandon’s Matrix, a theoretical framework from a philosopher’s account (Brandon 1994) to attain conceptual clarity about the characterisation of scientific methods. Subsequently we used Brandon’s Matrix as a tool to help us design a methodological approach and to investigate teachers’ understanding and views of the diversity of scientific methods. As such, the adaptation of Brandon’s Matrix constitutes a contribution to the theoretical framing and the methodological investigation of scientific methods in science education. If teachers are expected to teach a variety of scientific methods (NGSS Lead States 2013), they first and foremost need be able to recognise the scientific methods used in science.

The results of the present study showed that the majority of teachers answered correctly in both of the given scenarios. However, since Task 1 was developed to indicate teachers’ “baseline” understanding of scientific methods, these scores cannot be interpreted as satisfactory. It was expected that a greater number of teachers would choose the right answer, as the presented scenarios were exemplary investigations for the two ends of Brandon’s Matrix (manipulative hypothesis testing and non-manipulative parameter measurement). This finding provides a valuable insight on teachers’ conceptual understanding of scientific methods suggesting that there is a need for further investigation of the concept.

With regard to teachers’ views on scientific methods, the results showed that more than half of the sample hold naïve views on scientific methods. More specifically, most of the participants stated that scientific investigations follow a universal method that includes step-by-step procedures. Moreover, the findings show that the majority of the teachers view the manipulation of a variable or hypothesis testing as necessary components of scientific investigations. These findings are in line with findings from recent studies showing a sufficient, yet not satisfactory level of teachers’ views on scientific methods (e.g. Kartal et al. 2018).

Teachers’ naïve views on scientific methods can be explained by the traditional depiction of methods applied in science by means of “the scientific method”, which represents scientific methods as follows: (a) a linear and stepwise process, which involves (b) the manipulation of a variable and hypothesis testing (Woodcock 2014). Since teachers have widely used this paradigm to teach scientific methods, their views are likely to be influenced by the theoretical assumptions that it encompasses. Subsequently, teachers’ naïve views on scientific methods may explain their difficulties to teach a wider spectrum of scientific methods (Kampourakis 2016), as teachers would not be able to teach what they themselves do not understand (Bartos and Lederman 2014).

To further investigate patterns in teachers’ responses, inter-item correlations on items measuring teachers’ views on scientific methods were calculated showing a positive correlation between teachers’ views on variable manipulation and their views on hypothesis testing. Based on this finding, we conclude that the participants holding more informed views about scientific methods acknowledged that scientific investigations can take different forms and may include or not include manipulations and hypothesis testing.

Finally, the results presented a positive relationship between teachers’ overall understanding of scientific methods and their views on the how science operates. More explicitly, teachers who were able to identify the cases of hypothesis testing and variable manipulation within the given scenarios (Task 1), were more likely to hold more informed views on “what counts” as scientific investigation (Task 2). Although this finding may seem expected, it strengthens the argument for the integration of explicit instruction of the scientific methods in teacher training programmes. While past studies on teacher NOS views have focused on the measurement and improvement of teachers’ general perceptions on science, the results of the present study reveal that the teachers’ basic understanding of scientific methods should not be assumed a given.

Despite the pedagogical affordances of heuristics such as Brandon’s Matrix, teachers’ understanding of the scientific methods is a prerequisite for the adaptation of such tools in teaching practice. Thus, the question that arises is how well teachers themselves know how to identify scientific methods, although teachers’ views about NOS are likely to be influenced by their prior views about science (Akerson et al. 2009).

The results suggest that there is still room for improvement with regard to teachers’ understanding and views of scientific methods. While this finding in itself is not entirely surprising, what our analysis accomplishes is a tangible, specific and coherent manner in which teachers’ understanding of scientific methods can be investigated and supported. Teachers’ depiction of scientific methods using Brandon’s Matrix components actually points out where teachers’ understanding is limited and how such understanding can potentially be targeted in order to directly address the problem. Accordingly, specific teacher education interventions can be designed to be inclusive of relatively underspecified aspects of Brandon’s Matrix in order to broaden teachers’ understanding. Examples of effective interventions are available (Kaya et al. 2019) including their impact on pre-service teachers’ visual representations of scientific methods (Erduran and Kaya 2019; 2018). Furthermore, in order to support teachers’ understanding and teaching of scientific methods, our team has produced some resources teaching and summative assessment based on Brandon’s Matrix. These resources can be accessed at the project website (projectcalibrate.web.ox.ac.uk).

Our findings are in line with previous research suggesting that teachers’ NOS views are not informed (Capps and Crawford 2013) and that teachers endorse the idea of “the scientific method” (Woodcock 2014). The results provide insight into teachers’ misconceptions regarding “what counts as a scientific investigation”, which can serve as a starting point for teachers’ trainings on scientific methods. The study also suggests the use of heuristic frameworks, such as Brandon’s Matrix, as analytical tools for the development and the analysis of tasks. Overall, the study provides data on teachers’ background knowledge about scientific methods which is critical to understand if ultimately the students will be taught a balanced account on the diversity of scientific methods.

## Data Availability

Not applicable
